# Manual Therapy Research Methods in Animal Models, Focusing on Soft Tissues

**DOI:** 10.3389/fnint.2021.802378

**Published:** 2022-01-28

**Authors:** Geoffrey M. Bove, Susan L. Chapelle, Matthew J. S. Barrigar, Mary F. Barbe

**Affiliations:** ^1^Bove Consulting, Kennebunkport, ME, United States; ^2^Center for Translational Medicine, Lewis Katz School of Medicine, Temple University, Philadelphia, PA, United States; ^3^Canadian Memorial Chiropractic College, Toronto, ON, Canada

**Keywords:** manual therapy, massage, animal model, complementary - integrative - multidisciplinary - multimodal pain management, complementary & alternative medicine

## Abstract

Manual therapies have been practiced for centuries, yet little research has been performed to understand their efficacy and almost no animal research has been performed to inform mechanisms of action. The methods of manual therapy practice are quite varied and present a challenge for scientists to model the treatments and perform research using rodents. In this perspective we present a descriptive analysis of the complexity of the treatments, highlighting the role of tissue mechanics and physics. With these complexities in mind, we compare using manual therapy as clinically practiced, to attempts to develop machinery to model or mimic manual therapy. We propose that because of the complexities of manual therapy as practiced, having therapists perform the treatments on research animals just as they would on humans is the most scientific approach. Our results using this approach have supported its practicality.

## Introduction

Manual therapy is any treatment delivered by hands that carries a beneficial intent. There are numerous professions that perform various forms of manual therapy, which have rich histories that can be traced for centuries.^1^ Recent years have seen an upsurge of research into the effects and therapeutic mechanisms of manual therapy. Biomedical scientists have partnered with manual therapists to develop approaches to mechanistically study therapies. Which approaches are most relevant and best suited for translation into clinical care? Here we offer our perspective on animal-based manual therapy research methods, with the goal to persuade the reader that simple direct methods translation is the preferable approach.

This perspective will be limited to manual therapy performed for conditions that are thought to have a pathology in the area being treated. Treatment for painful conditions, including those without identified pathologies in the area being treated, is the most common reason for seeking manual therapy. Manual therapy is also sought for relaxation and overall health benefits; while we are not diminishing these possible benefits, we are not including this in our perspective. We are also not including joint manipulation since this is a special skill and enjoys a well-developed research base. This perspective applies to the application of forces with the intent of compressing and moving structures in relationship to other structures, generally referred to as mobilization.

## Discussion

There are two general approaches to delivering manual therapy to animals. A primary decision for the investigator is whether to deliver the manual therapy using hands or using a machine. For both approaches, the applied forces are considered critical.

Manual therapy is performed on unanesthetized humans, and therefore from the start, we applied manual therapy using our hands on unanesthetized rats and mice. Our methods are dictated by vast experience with manual therapy on humans, and also by extensive experience handling a variety of laboratory and pet animals. The most common laboratory animals are rats and mice. Rats are amenable to being held, and after accommodation, often seek out human physical interaction (Davis and Pérusse, 1988; Reinhold et al., 2019). Mice are far less sociable with humans, require “scruffing” to be handled, and their small size makes them less desirable for manual therapy studies.

There are numerous advantages to this approach:

1. It emulates the clinical setting, and therefore is immediately translatable.
2. The forces are determined and constantly modified by the operator, as in the clinic.
3. It is more humane. Awake animals (like humans) will respond by flinching or other behavior if the treatment is too intense. Anesthesia is safe, but induction is stressful to animals, and we do not know what they perceive during recovery (such as headache or nausea).
4. It is easier, simpler, and faster.
5. It does not require the design and manufacture of special devices that cannot replicate human palpation skills.

While we have not discovered impediments, there is a primary perceived drawback in that that we cannot precisely quantify the forces that are delivered. This concern is commonly raised by colleagues who have no experience in performing manual therapy, which includes most scientists. We need to consider physics, anatomy, and biomechanics to address this concern.

In terms of physics, a dose of manual therapy is the total amount of energy delivered to the tissues, called *power*. The therapist provides forces (stresses), and the patient provides resisting forces, necessary to create power. The forces are dissipated by the patient by displacements and elastic strain. However, therapists and patients present too many variables to make quantification possible in a meaningful fashion:

1. The size, shape, and density of the parts of the therapists that are used to provide forces vary greatly. This can be confirmed by comparing different fingertips and hands. Thus, the same force from different therapists will result in different pressures (pressure = force/area; N/m^2^), and these pressures will vary based on the profile of the part being used. When a force is delivered by a small applicator (such as a thumb), the contact profile and area change as the force increases, which varies the pressure. Finally, the delivery angle usually varies during treatments to get around or under anatomical parts, also modifying the force.

2. Patients vary greatly in size, shape, and tissue proportion. The absorption and dissipation of delivered forces are dictated by anatomy and tissue biomechanics. Each type of structure (e.g., skin, fat, muscle, bone, and tendon) has a different consistency in terms of density and viscoelastic behavior, and is almost always found together, making our structures biomechanically complex. Muscle consistency (tone) and size (volume), and the amount and consistency of the skin and subcutaneous fat, vary greatly between individuals. The interfaces between tissues are essentially frictionless, allowing structures to slide to allow movements.

Because of these differences, forces (stresses) applied to soft structures are variably dissipated and absorbed (strained). Neither are standardized in clinical practice; in other words, there is no consistent force application method to model for treatment standardization. Since modeling manual therapy is subject to these variables, we maintain that attempting to standardize treatment parameters in the laboratory becomes fallacious as soon as one of the parameters is overlooked, such as applying a force without considering the pressure. Many of these challenges were acknowledged in recent reviews of deep tissue and Tui na massage methods (Fang and Fang, 2013; Koren and Kalichman, 2018).

The reader can appreciate these challenges by performing a brief exercise (it is helpful to have an anatomy book available to help visualize the structures). Gently press into the center of the forearm with your first and middle fingertips and slide them from side to side. The skin should slide freely over the muscle compartment, allowing you to feel harder and softer structures that are deeper in the arm. Press straight in and you may feel the structures spread. There will be a point where the movements will lessen, and the resistance will increase sharply. This occurs when the structure is compressed to its limit by being pressed against the fascial constraints of itself or neighboring structures or pinched between the skin and bone. In physics terms, this is when the combined elastic limit of the structures is reached. Next, press more gently into your forearm near the wrist, and feel the ropey tendons. Shift your attention to your neck just below your ear and compare the texture and mobility of the structures. Then find a consenting person to perform the same procedures upon. Pay attention to the way the structures move, and when you sense resistances.

This simple exercise should be convincing that efforts to reproduce the force profiles of a manual therapy treatment are not simple. There is no place in the human body where the various tissues remain in the same proportion or dimension and thus feel the same. This is important because applied forces will lead to very different strains of the structures. These factors conspire to dictate how the target structure responds, and how it changes its relationship to other structures. In a laboratory setting, applying consistent stress in a single plane is feasible, but maintaining the control while moving would add complexity, as would varying the angle and other parameters. We contend that such efforts are unnecessary, and undermine the importance of the therapist.

These treatment complexities are taken into consideration by therapists, who continuously monitor the structures being treated, consciously and unconsciously. Manual therapy methods have been taught based on collective experience, without the benefit of science. It should not be surprising that manual therapy training varies greatly, with therapeutic forces being very light in some methods and quite heavy in others. Therapists alter which part of their fingers or hands to apply the force with, meaning that they are continuously controlling and modifying the pressure (force/area), depending on their goals. A smaller finger will go deeper, either to press on a perceived “knot” or with the idea of separating a fascial plane. A palm will apply pressure over a broader area. Structures will often be grasped and moved over underlying structures. Therapists use feedback from the patient and from the structures to dictate how much pressure to use, and often leave and then return to a sensitive area. A cognitive response arises from the therapist to modulate pressure in response to sensing a change in tissue resistance that may have to do with a combination of viscoelastic adaptation, focal neural feedback, central neural feedback (like the patient or animal relaxing), and focal vascular and lymphatic responses. These cognitive responses from therapists are a response to dynamic processes between the therapist and the patient or animal. Therapists scale their treatments to accommodate the size and shape of their patient and the clinical presentation, which may include sensitized and diseased tissues, and scaling to a rodent has not presented any challenge.

It follows that investigating the pressures delivered by manual therapies is a highly complex undertaking. The first challenge would be to map the reaction (e.g., internal pressure, displacement, deformation) of various structures (e.g., arm, leg, back; tendon, nerve, muscle, and fat within and surrounding structures) to device-delivered forces, while varying applicator size and angles. The results could potentially be expressed as a complicated dose, which would still have to be characterized using vectors and time. The same process would have to be performed with rodent structures, to allow scaling. Machines would have to be constructed to provide forces through probes with varied dimensions, at variable angles, under feedback control. The feedback would have to include factors that we have described, which are not fully tangible. While not impossible, we are concerned that this would serve no practical purpose, at least not until we have evidence that the methods are clinically effective and there are justifiable concerns that different methods have different efficacy.

**Figure 1:**
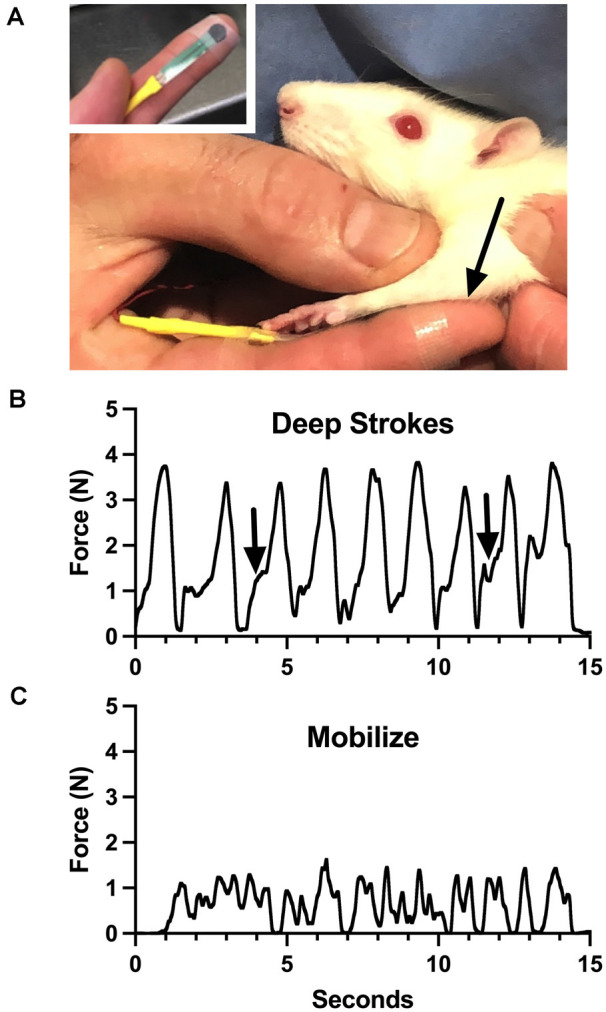
Training device for manual therapy (modified from Barbe et al., 2021). **(A)** A small pressure-sensitive resistor powered by a bridge amplifier was attached to the therapist’s finger and used to monitor force applied through it to a rat upper limb (arrow). **(B)** Output during “deep strokes” as depicted in **(A)**. Arrows indicate skewness of application cycles, where the skin was displaced cephalad to allow deeper penetration without pulling fur. **(C)** Output during side-side mobilization of the flexor compartment of the arm. See text for more details. Figure 1 reproduced from Barbe et al. (2021) under the Creative Commons Attribution License (https://creativecommons.org/licenses/by/4.0/legalcode).

Aware of these limitations, we have attempted to roughly characterize the general force profiles of the treatments we provide, as a potential training tool. Using a flexible force-sensitive resistor applied to a treating finger, we performed some components of the treatment we give to rats (Figure 1, reproduced from Barbe et al., 2021; also see Bove and Nilsson, 1999). The device responds to forces generated perpendicular to the surface (called “normal”). We made some interesting observations. Even calibration of the device required assumptions, including which substrate to use to provide resistance (we used a soft silicone pad). We had to accept many variables, including finger size differences, bending, and the inconsistent area of coverage of the device during the dynamic treatment, all of which determine the output of the system. These make the force recordings imprecise, and even misleading. The lead author generated the force profiles. Potential therapists-in-training used the device to practice treatment intensity and timing while referring to the force profiles. The force profiles reveal many of the limitations discussed above. For instance, the “deep strokes,” which resemble the most common massage maneuver, are the most easily captured force since they are applied normal to the surface. Note however that each force is skewed to the left (Figure 1B, arrows), which reflects the technique: after contact, the skin of the rat is displaced cephalad, followed by an increase of force as the finger is pressed into the forelimb and dragged caudad. None of this is explained by the force trace, and the strains are not measured. Also, the beginning of each stroke was in the large flexor compartment of the sharply tapered arm, while the end of the stroke was in the smaller distal part of the arm. Figure 1C shows the output of the sensor during mobilization, where the flexor muscles were grasped between the thumb and the finger and slid back and forth over the underlying structures. The force profile here has no relevance to the shear forces, which are putatively the important part of this treatment, which were delivered 90 degrees to the forces represented by the graph. While this tool seemed somewhat effective for training inexperienced persons to provide the treatment, we concluded that it was better to employ a therapist with formal training and experience with manual therapy in humans (also see below).

There have been a few reports where mechanical loading was applied to anesthetized animals using devices. Butterfield et al. developed a device to perform cyclical compressive loading, which they termed a “massage mimetic” (Butterfield et al., 2008). This laboratory has been very productive, revealing important effects and mechanisms of the treatment (Waters-Banker et al., 2014; Hunt et al., 2019). As a starting point, the forces to be used in the rabbit tibialis anterior were scaled to the human paraspinal muscles. Although the device controls the applied force and measures the applied force at the surface, it cannot measure the response of the tissues. The treatment does not resemble manual therapy, where the therapist’s fingers would seek the interspaces beside the muscles and apply very different forces as the structures tapered. The dose cannot be determined using this method because of the factors listed above. More recently, a computer-controlled robotic device was developed to deliver quantified mechanotherapy to rodents (Seo et al., 2021). While more sophisticated by virtue of feedback for force control and strain modeling, results from the use of this device are subject to considerations detailed above, starting with the assumption that the structure treated was homogenous (bone and muscle) and by not considering the delivery angle. These methods are providing mechanical loading, but they are not delivering manual therapy. Although this does not diminish the importance of their findings, it makes clinical translation cumbersome, unless the goal is to develop devices to perform mechanotherapy rather than to employ trained therapists.

Our route has been far simpler. We took advantage of our collective and extensive therapeutic experience to scale treatments to the small structures of the rat (limbs and abdomen). We tested and refined the methods on normal rats, rats that had undergone abdominal surgery (Chapelle and Bove, 2013; Bove et al., 2017), and rats that had developed advanced repetitive motion disorders (Bove et al., 2016). We only used treatments that were consistent with common clinical practice and that were readily accepted with no adverse reactions by the rats. Treatment components were tested on multiple rats, and primarily involved scaling [some components can be viewed in Bove et al. (2019), supplementary data]. An example of a refinement included placing a 4.5 mm metal ball under a glove to perform a “deeper” treatment to a rat forearm. This was met with resistance of the awake rats, indicating that it was too much focal pressure, and was therefore not included in our treatment protocols (Bove et al., 2016, 2019; Barbe et al., 2021).

We have had numerous therapists from different fields (massage therapy, chiropractic, physical therapy, and speech pathology) handle and treat rats. All have readily performed the methods and reported that the treatments are reasonable and similar to working on humans. Since the treatments were never deconstructed, they did not have to be reconstructed to be understandable to the therapists providing the treatments; this is critical to our argument. Therefore, while highly complicated if broken down into components for the laboratory, the dose we use seems readily portable to experienced therapists, and thus to the professions that perform manual therapy on humans.

We have also learned in our studies that the relationship between the rat and the person performing the manual therapy is important. While there are differences in the cooperation of individual rats, the approach of the person performing the manual therapy is also important. It is critical that the therapist be confident. Readers familiar with performing behavioral studies in rats (such as testing paw sensitivity) know that results vary as much by the operator as they do by the rat. Thus, using the same person for all studies is standard practice. We have also concluded that experienced therapists should provide the therapy since some skills that are automatic for an experienced therapist must be taught to a novice. Examples of such skills include always maintaining awareness of the contact with the animal to prevent pulling the hair, and the gentle following of the rat as it moves rather than restraining, thus preventing struggling. Although we have not observed any differences between male and female therapists, we suggest that until a direct comparison has been made, the same therapist is used within any experiment.

We propose that the immediate future of manual therapy research focuses on establishing efficacy for specific disorders and to determine what pathologies are affected by manual therapy. Therapists seem to universally agree with the statement “movement good, stasis bad,” implying that they can feel that something is not moving normally but should be addressed. From our studies in rodent models, we know that manual therapy prevents chronic fibrosis and inflammation, along with the symptoms consistent with pain and dysfunction. Similar human studies are possible but have not been performed. Efficacy against a pathology seems necessary for the expansion of manual therapy into medical settings, where it is not currently used for disorders, but almost exclusively for symptomatic treatment. We maintain that research is unlikely to change the treatments themselves, which are ensconced. We need to appreciate that treatments are guided by clinical impressions, but that we have not yet determined what the therapists are feeling to guide them to deliver a “dose.” Treatment quantification can be holistic and still be used to demonstrate clinical efficacy, as it has done for chiropractic care (Meade et al., 1995).

While the interest in manual therapy research represents great progress, we now need to partner with and learn from the hundreds of thousands of therapists worldwide to make sure our efforts remain relevant to their practice. Finally, we urge that scientists personally experience manual therapy and practice its basics on numerous people prior to undertaking scientific studies that involve manual therapy.

## Data Availability Statement

The original contributions presented in the study are included in the article, further inquiries can be directed to the corresponding author.

## Author Contributions

GB conceptualized this manuscript and wrote the first draft. All other authors made critical additions and approved the submitted version.

## Conflict of Interest

The authors declare that the research was conducted in the absence of any commercial or financial relationships that could be construed as a potential conflict of interest.

## Publisher’s Note

All claims expressed in this article are solely those of the authors and do not necessarily represent those of their affiliated organizations, or those of the publisher, the editors and the reviewers. Any product that may be evaluated in this article, or claim that may be made by its manufacturer, is not guaranteed or endorsed by the publisher.

## Footnotes

^1^It is not within the scope of this article to present this historical information.
